# Publisher Correction: Mediator complex interaction partners organize the transcriptional network that defines neural stem cells

**DOI:** 10.1038/s41467-019-11254-1

**Published:** 2019-07-22

**Authors:** Marti Quevedo, Lize Meert, Mike R. Dekker, Dick H. W. Dekkers, Johannes H. Brandsma, Debbie L. C. van den Berg, Zeliha Ozgür, Wilfred F. J. van IJcken, Jeroen Demmers, Maarten Fornerod, Raymond A. Poot

**Affiliations:** 1 Department of Cell Biology, Erasmus MC, Wytemaweg 80, 3015 CN Rotterdam, Netherlands; 2 Center for Proteomics, Erasmus MC, 3015 CN Rotterdam, Netherlands; 3 Center for Biomics, Erasmus MC, 3015 CN Rotterdam, Netherlands

**Keywords:** Biochemistry, Gene regulation, Chromatin structure, Transcription, Neural stem cells

Correction to: *Nature Communications* 10.1038/s41467-019-10502-8, published online 17 June 2019.

The original version of this Article contained an error in the spelling of the author Wilfred F. J. van IJcken, which was incorrectly given as Wilfred F. J.van IJcken. This has now been corrected in both the PDF and HTML versions of the Article.

