# Supplementary material for: Mediator complex interaction partners organize the transcriptional network that defines neural stem cells
Source: Nat Commun. 2019 Jun 17;10:2669. doi: 10.1038/s41467-019-10502-8 (PMC6573065; doi:10.1038/s41467-019-10502-8)
Supplement: Supplementary file 1 — Supplementary Information [file 41467_2019_10502_MOESM1_ESM.pdf]

Supplementary Information

Mediator complex interaction partners organize the transcriptional network that defines neural stem cells

Raymond A. Poot, et al.

Table of contents

Supplementary Figures

Supplementary Figure 1: Mediator complex and its interactors at promoters. (PDF)

Supplementary Figure 2: Tcf4 and its interactors at super enhancers and broad H3K4me3 promoters. (PDF)

Supplementary Figure 3: Human neural stem cells stain positive for Nestin and Sox2 (PDF)

Supplementary Figure 4: Uncropped gel of Figure 1b and westerns of Figure 2a,b. (PDF)

Supplementary Data

Supplementary Data 1: Interacting proteins of the Mediator complex, as identified by mass spectrometry analysis of FLAG-Med15 purifications. (xlsx)

Supplementary Data 2. Overlapping interacting proteins of the Mediator complex in Med12 immunoprecipitation, as identified by mass spectrometry analyses. (xlsx)

Supplementary Data 3: Super enhancers in NSCs and nearest active genes. (xlsx)

Supplementary Data 4: Gene Ontology terms of active genes in NSCs nearest to a super enhancer (SE) and/or having a broad H3K4me3 promoter. (xlsx)

Supplementary Data 5: Genes nearest to an SE and with broad H3K4me3 promoters in NSCs. (xlsx)

Supplementary Table 1: Data sets used in the study

Supplementary Table 2: Primers used in the study.

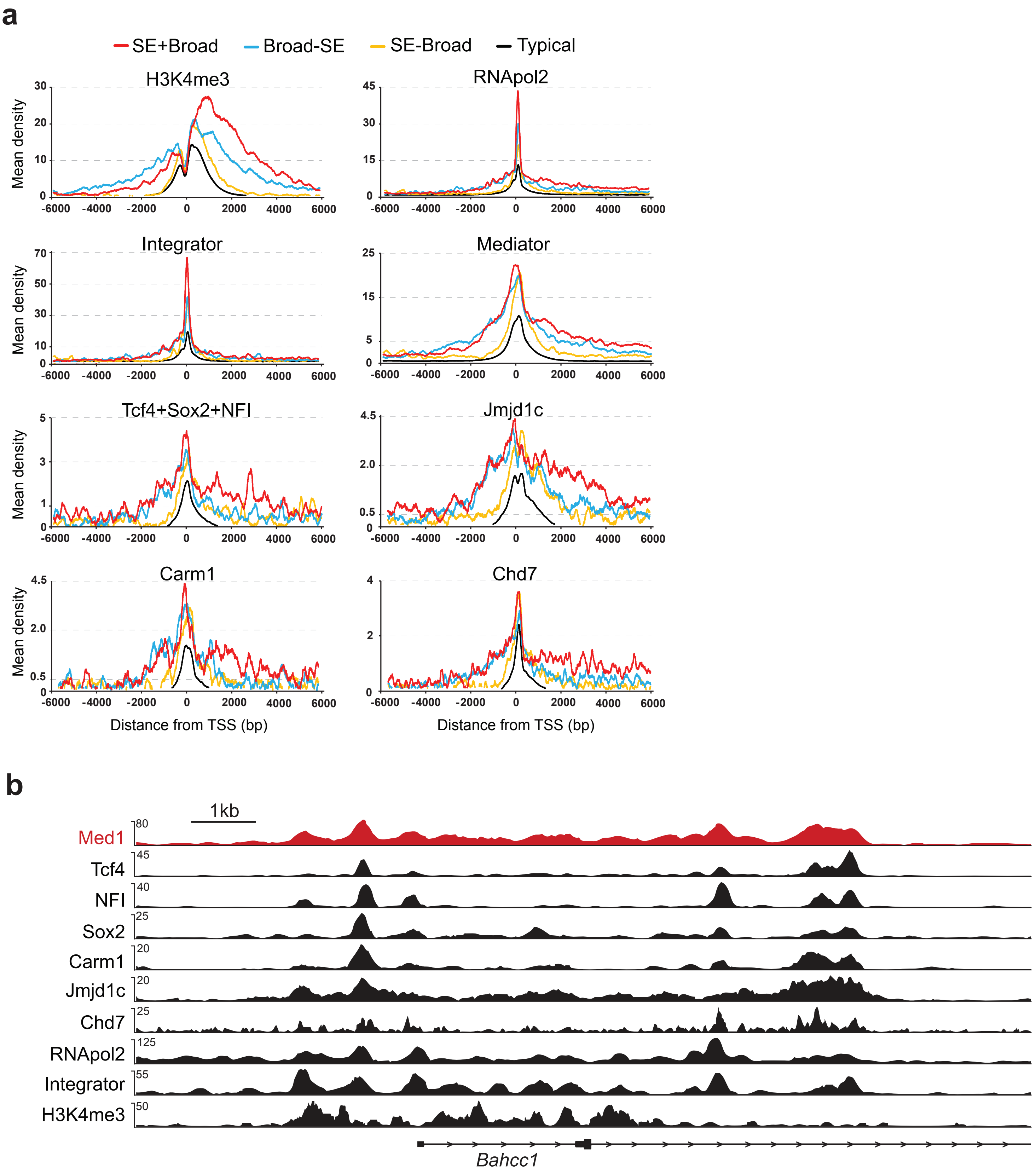

Supplementary Fig. 1 Mediator complex and its interactors at promoters.
(**a**) ChIP-seq density plots around top 100 promoters of Broad+SE genes, top 100 Broad-SE genes, top 100 SE-Broad genes and all typical genes for the indicated factors and histone modifications. Mean ChIP-seq density (y-axis) and distance to TSS (x-axis) are shown.
(**b**) Overlap of binding sites of Med1 with binding sites of Mediator interactors at the *Bahcc1* broad H3K4me3 promotor area NSCs. ChIP-seq tracks for the indicated proteins and histone modifications at the *Hes1* gene are shown. Range of reads per million per base pair is indicated on the y-axis. Scale bar is indicated.

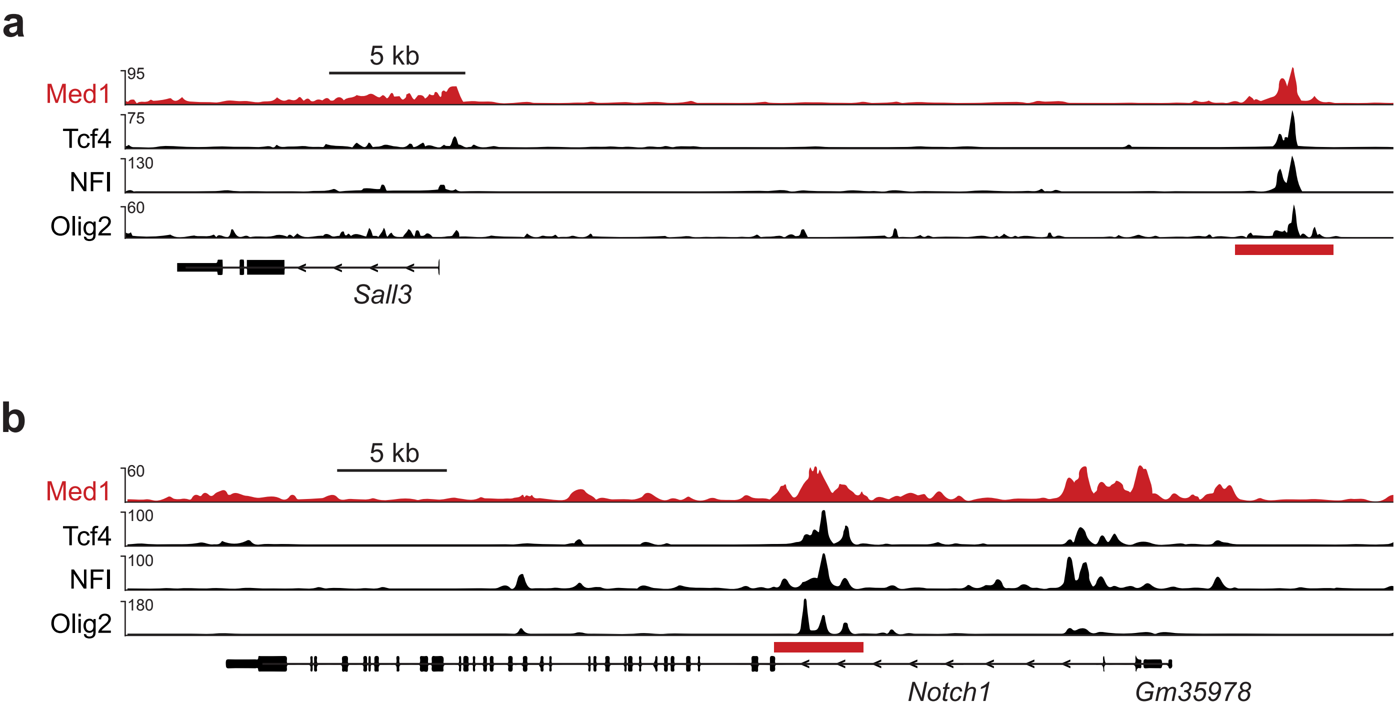

Supplementary Fig. 2 Tcf4 and its interactors at super enhancers and broad H3k4me3 promoters.

(**a**) Overlap of binding sites of Tcf4 and Med1 with binding sites of Tcf4-interactors Olig2 and NFI at the *Sall3* locus in NSCs. ChIP-seq tracks for the indicated proteins at the *Sall3* gene are shown. The *Sall3* SE is indicated with a red bar. Range of reads per million per base pair is indicated on the y-axis. Scale bar is indicated.

(**b**) Overlap of binding sites of Tcf4 and Med1 with binding sites of Tcf4-interactors Olig2 and NFI at the *Notch1* locus in NSCs. ChIP-seq tracks for the indicated proteins at the *Notch1* gene are shown. The *Notch1* SE is indicated with a red bar. Range of reads per million per base pair is indicated on the y-axis. Scale bar is indicated.

a

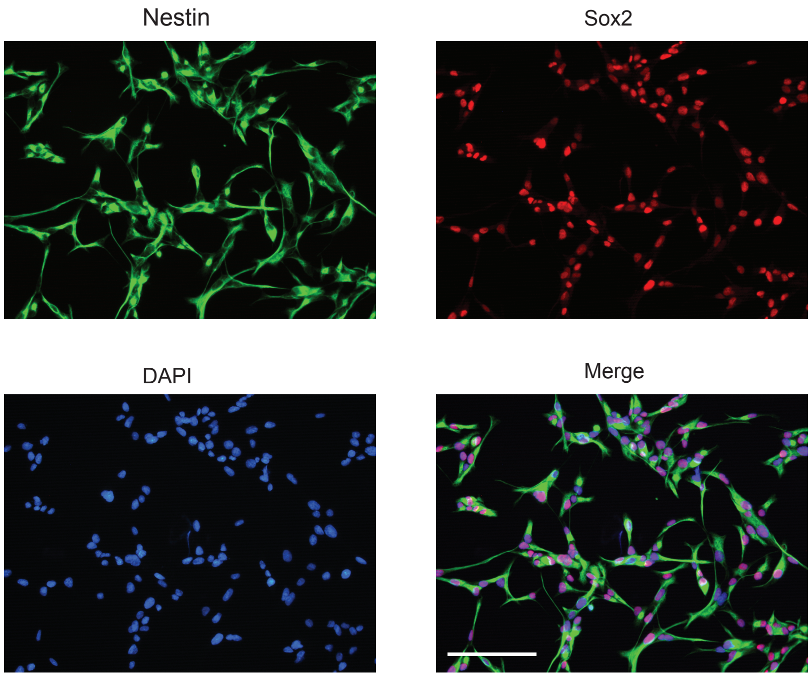

b

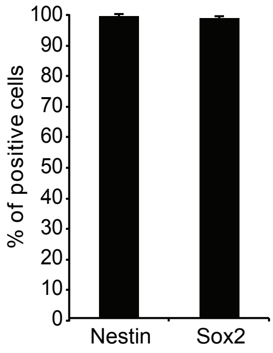

Supplementary Fig. 3 Human neural stem cells stain positive for Sox2 and Nestin.
(**a**) Staining of human neural stem cells with Nestin antibodies (left-upper panel), Sox2 antibodies (right-upper panel), DAPI (left-lower panel) and the merge of all stainings (right-lower panel). Bar indicates 100 µm.
(**b**) Percentage of human neural stem cells that stain positive for Nestin or Sox2. Percentage of all cells that stain positive for Nestin or Sox2 is indicated. S.e.m. of 6 technical replicate counts of 200 cells each on separate coverslips (1200 cells counted in total) is indicated.

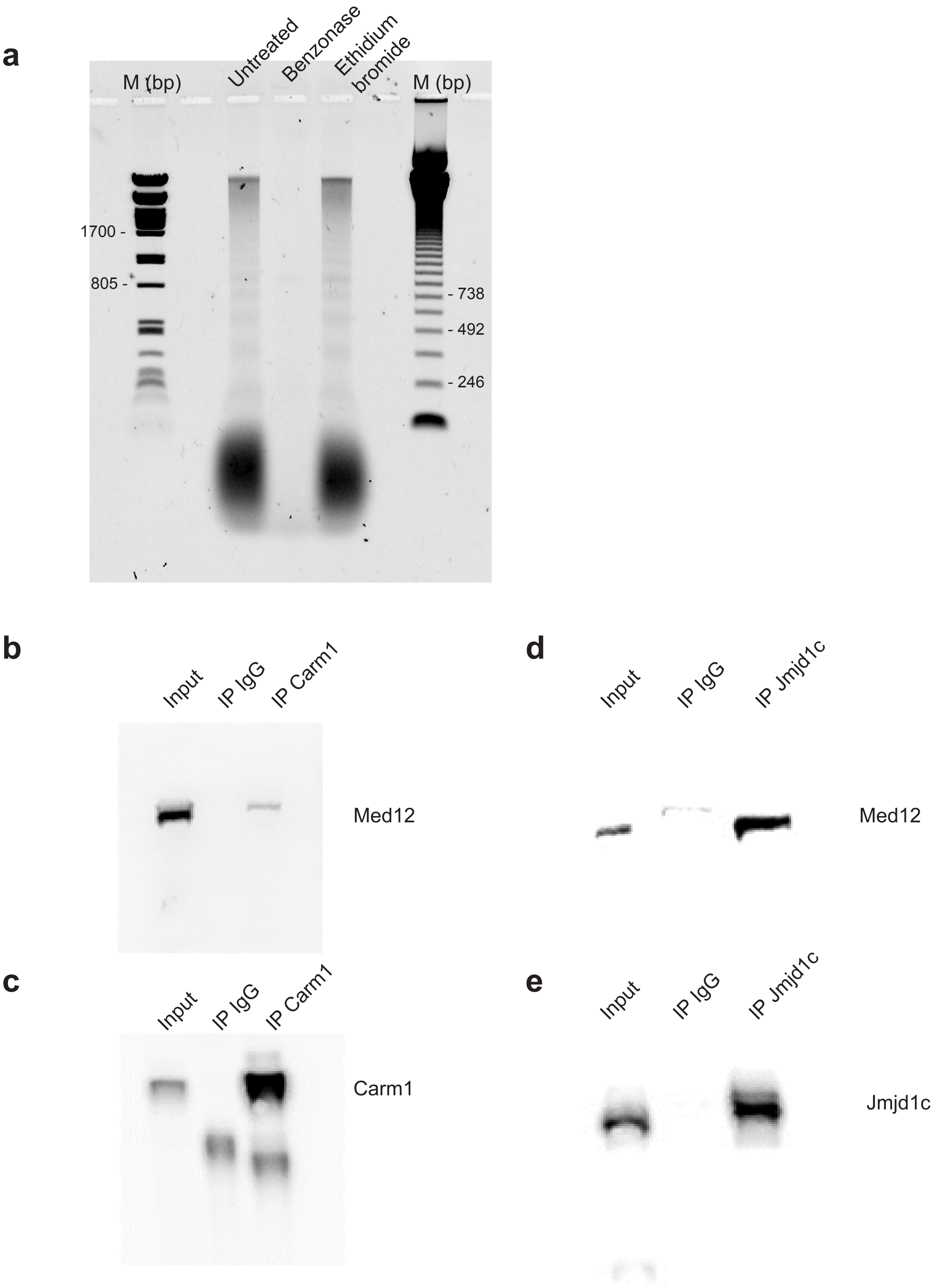

Supplementary Fig. 4 Uncropped gel and westerns.

(**a**) Uncropped DNA agarose gel of Figure 1b. Samples and DNA molecular weight markers are indicated.

(**b-e**) Uncropped western blot scans of Figures 2a and 2b. Samples are indicated on the top of the western panels, antibodies are indicated to the right of the corresponding westerns.

Supplementary Table 1. Data sets used in the study.

**ChIP-seq**	
Med1	GSM2928425
Jmjd1c	GSM2928426
Carm1	GSM2928427
IgG	GSM2928428
mNSCs Input	GSM1187180
Tcf4	GSM1820990
Sox2	ERR414096
NFI	ERR414099
Olig2	GSM1820994
Ints11	ERR1173522
RNA pol II	ERR1173526
Ep300	ERR216112
H3K27ac	ERR216108
H3K4me3	SRR006888_SRR006889
Cbx8	GSM2393587
Ctcf	GSM883647
**RNA-seq**	
wild-type NSCs	GSM1861892, GSM1861893, GSM1861894
scrambled shRNA NSCs	GSM1861886, GSM1861887, GSM1861888
Tcf4 knock down NSCs	GSM1861889, GSM1861890, GSM1861891

Supplementary Table 2. Primers used in the study.

Gene name or region	Use in qPCR	Sequence Fw	Sequence Rv
B-Actin	mRNA	5'- TCT CCT TCT GCA TCC TGT CAG CAA -3'	5'- TCT TGG GTA TGG AAT CCT GTG GCA -3'
Tcf4	mRNA	5'- AGT CTG CAA CTT CCC CTG AC -3'	5'- GTC GTC GGA TTT GAT CTC AG -3'
Sox2	mRNA	5'- AAA CAT GGC AAT CAA ATG TC -3'	5'- TTG CCA GTA CTT GCT CTC AT -3'
Amylase	ChIP	5'- CTC CTT GTA CGG GTT GGT -3'	5'- AAT GAT GTG CAC AGC TGA A -3'
Olig1 +30kb	ChIP	5'- GAC AGA AAG CAA CCA AGC AG -3'	5'- CAA GGA GAA AGG CAC TGA CA -3'
Olig1 -6,7kb	ChIP	5'- TTG CAT TCT GCC TGT TCA AT -3'	5'- CTT GCT GGA TGT TCC AAA TG -3'
Tulp3 +6kb	ChIP	5'- GTG TGA GCT GGA TTC TTC AG -3'	5'- GAC AGG AAA TGA CTC CTG GT -3'
Klf15 +8,6kb	ChIP	5'- CAG GGA GGT AAG AGG CCA TA -3'	5'- CTC CCA GGA AAG CAG AGA AC -3'
Jag1 +6,5kb	ChIP	5'- GAG TTG GCT GGA CTG ACT GA -3'	5'- ATC CTG AGA ATG TCC CGA GT -3'
