# Supplementary material for: Mediator complex interaction partners organize the transcriptional network that defines neural stem cells
Source: Nat Commun. 2019 Jun 17;10:2669. doi: 10.1038/s41467-019-10502-8 (PMC6573065; doi:10.1038/s41467-019-10502-8)
Supplement: Supplementary file 3 — Description of Additional Supplementary Files [file 41467_2019_10502_MOESM3_ESM.pdf]

Description of Additional Supplementary Files

File name: Supplementary Data 1

Description: Interacting proteins of the Mediator complex, as identified by mass spectrometry analyses of FLAG-Med15 purifications. (supplied as xlsx file)

Purifications from benzonase-treated nuclear extracts (benzo), non-treated nuclear extracts and Ethidium Bromide-treated nuclear extracts (EtBr) and a control purification are shown. In addition, a replicate purification from benzonase-treated nuclear extract and a replicate control purification is shown. Per protein, numbers of unique peptides detected by mass spectrometry per experiment are shown, emPAI scores are shown and Mascot scores are shown.

File name: Supplementary Data 2

Description: Overlapping interacting proteins of the Mediator complex in Med12 immunoprecipitation, as identified by mass spectrometry analyses. (supplied as xlsx file)

Med12 antibody immunoprecipitations and IgG control purifications from untreated nuclear extracts (No benzo) and benzonase-treated nuclear extracts (benzo) are shown. Proteins that overlap with FLAG-Med15 purifications (Supplementary Table 1) are shown. Per protein, numbers of unique peptides detected by mass spectrometry per experiment are shown, emPAI scores are shown and Mascot scores are shown. Proteins absent are highlighted in grey, proteins not sufficiently enriched in Med12 IPs over control IPs are highlighted in red.

File name: Supplementary Data 3

Description: Super enhancers in NSCs and nearest active genes. (supplied as xlsx file)

Chromosomal positions of super enhancers are shown, nearest active gene and super enhancer (SE) ranking are shown.

File name: Supplementary Data 4

Description: Gene Ontology terms of active genes in NSCs nearest to a super enhancer (SE) and/or having a broad H3K4me3 promoter. (supplied as xlsx file)

Left-upper panel; Gene Ontology (GO) terms genes and transcription factor genes (TF genes) with a broad H3K4me3 promoter. Middle upper panel; GO terms genes and TF genes nearest to an SE. Right-upper panel; GO terms genes and TF genes nearest to an SE and with a broad H3K4me3 promoter. Left-bottom panel; GO terms genes and TF genes with a broad H3K4me3 promoter but not nearest to an SE. Middle-bottom panel; GO terms genes nearest to an SE but without broad H3K4me3 promoter.

File name: Supplementary Data 5

Description: Genes nearest to an SE and with a broad H3K4me3 promoter in NSCs. (supplied as xlsx file)
