# Supplementary material for: Mediator complex interaction partners organize the transcriptional network that defines neural stem cells
Source: Nat Commun. 2019 Jun 17;10:2669. doi: 10.1038/s41467-019-10502-8 (PMC6573065; doi:10.1038/s41467-019-10502-8)
Supplement: Supplementary file 9 — Reporting Summary [file 41467_2019_10502_MOESM9_ESM.pdf]

nature research

Corresponding author(s): Raymond Poot

Last updated by author(s): 01-05-2019

Reporting Summary

Nature Research wishes to improve the reproducibility of the work that we publish. This form provides structure for consistency and transparency in reporting. For further information on Nature Research policies, see Authors & Referees and the Editorial Policy Checklist.

Statistics

For all statistical analyses, confirm that the following items are present in the figure legend, table legend, main text, or Methods section.

n/a	Confirmed	
☐	☒	The exact sample size (*n*) for each experimental group/condition, given as a discrete number and unit of measurement
☐	☒	A statement on whether measurements were taken from distinct samples or whether the same sample was measured repeatedly
☐	☒	The statistical test(s) used AND whether they are one- or two-sided<br>*Only common tests should be described solely by name; describe more complex techniques in the Methods section.*
☒	☐	A description of all covariates tested
☒	☐	A description of any assumptions or corrections, such as tests of normality and adjustment for multiple comparisons
☐	☒	A full description of the statistical parameters including central tendency (e.g. means) or other basic estimates (e.g. regression coefficient) AND variation (e.g. standard deviation) or associated estimates of uncertainty (e.g. confidence intervals)
☒	☐	For null hypothesis testing, the test statistic (e.g. *F*, *t*, *r*) with confidence intervals, effect sizes, degrees of freedom and *P* value noted<br>*Give P values as exact values whenever suitable.*
☒	☐	For Bayesian analysis, information on the choice of priors and Markov chain Monte Carlo settings
☒	☐	For hierarchical and complex designs, identification of the appropriate level for tests and full reporting of outcomes
☒	☐	Estimates of effect sizes (e.g. Cohen's *d*, Pearson's *r*), indicating how they were calculated

Our web collection on statistics for biologists contains articles on many of the points above.

Software and code

Policy information about availability of computer code

Data collection	Commercial code: Excel 2010, no privaye code used
Data analysis	Commercial code: Mascot Distiller 2.1, Mascot search algorithm 2.2, EmPAI, Bowtie v. 12.7, MACS46 v1.4.2, Homer Findpeaks, ROSE, DAVID v6.7. No private code used

For manuscripts utilizing custom algorithms or software that are central to the research but not yet described in published literature, software must be made available to editors/reviewers. We strongly encourage code deposition in a community repository (e.g. GitHub). See the Nature Research guidelines for submitting code & software for further information.

Data

Policy information about availability of data

All manuscripts must include a data availability statement. This statement should provide the following information, where applicable:

- Accession codes, unique identifiers, or web links for publicly available datasets
- A list of figures that have associated raw data
- A description of any restrictions on data availability

The accession number for the genomic data reported in this paper is GEO: GSE109043. The mass spectrometry proteomics data have been deposited to the ProteomeXchange Consortium via the PRIDE partner74 repository with the dataset identifier PXD013546.
Figure 1c and Supplementary Tables 1, 2 and 3 have associated proteomic data. Figures 2d, 3, 4, 6 and 7, Supplementary Figures 1 and 2, Supplementary Tables 4 and 5 have associated genomic data. The source data underlying Figures 1b, 2a and 2b, 3a and 3c, 5a-d, 6c and 7c are provided as a Source Data file. There are no restrictions on data availability.

Field-specific reporting

Please select the one below that is the best fit for your research. If you are not sure, read the appropriate sections before making your selection.

☒ Life sciences ☐ Behavioural & social sciences ☐ Ecological, evolutionary & environmental sciences

For a reference copy of the document with all sections, see nature.com/documents/nr-reporting-summary-flat.pdf

Life sciences study design

All studies must disclose on these points even when the disclosure is negative.

Sample size	Biological triplicates are common practise for RNA-seq experiments (Fig. 3c, Fig. 6c, Fig. 7c).<br>Biologically independent duplicates are common practise for ChIP experiments (Figure 5)
Data exclusions	No samples were excluded from the experiments.
Replication	FLAG-Med15 IPs were performed four times, confirmatory Med12 antibody IPs were performed twice. ChIP-seq for Med15, Carm1 and Jmjd1c were performed once and confirmed by ChIP on individual genes for Mediator in duplicate. Staining for NSC markers were performed in technical triplicates. The used replication are within standards in the field. All attempts at replication were successful.
Randomization	Not relevant
Blinding	Not relevant

Reporting for specific materials, systems and methods

We require information from authors about some types of materials, experimental systems and methods used in many studies. Here, indicate whether each material, system or method listed is relevant to your study. If you are not sure if a list item applies to your research, read the appropriate section before selecting a response.

Materials & experimental systems

n/a	Involved in the study	
☐	☒	Antibodies
☐	☒	Eukaryotic cell lines
☒	☐	Palaeontology
☒	☐	Animals and other organisms
☒	☐	Human research participants
☒	☐	Clinical data

Methods

n/a	Involved in the study	
☐	☒	ChIP-seq
☒	☐	Flow cytometry
☒	☐	MRI-based neuroimaging

Antibodies

Antibodies used	goat anti-Sox2 (Santa-Cruz Biotechnology sc-17320,) Rabbit anti-Nestin (Biolegend® 839801), Med12 antibody (Bethyl Laboratories #A300-774A), Jmjd1c antibody (Merck Millipore #17-10262), Carm1 antibody (Cell Signaling Technology #12495), Med1 (Bethyl Labs #A300-793A), IgG (Normal Rabbit IgG: Santa Cruz #sc-2027).
Validation	All antibodies have been successfully used by other labs for the purpose that we used them. Each antibody recognizes one protein band on of the correct size on a western on the supplier's website, strongly indicating that the correct protein is recognized. All proteins in the paper are initially identified to interact by mass spectrometry, antibodies are just used to confirm these interactions and validate that interacting proteins also colocalize on the genome. The Sox2 and Nestin antibodies used as markers for NSCs are used numerous times by other labs for that purpose and Sox2 and Nestin have been confirmed to be NSC markers by other methods.

Eukaryotic cell lines

Policy information about cell lines

Cell line source(s)	NS-5 neural stem cells (NSCs) were derived from 46C embryonic stem cells and cultured on N2B27 medium (Stem Cell Sciences) supplemented with EGF and FGF (both from Peprotech) and regularly tested for mycoplasma contamination.
Authentication	46C ESCs can be recognized by GFP in the Sox1 locus, which switches on upon neural differentiation. 46C NSCs were stained with NSC markers Sox2 and Nestin (Supplementary Fig. 3a and 3b).

Mycoplasma contamination	NSCs are regularly tested for mycoplasma contamination.
Commonly misidentified lines (See ICLAC register)	None used.

ChIP-seq

Data deposition

☒ Confirm that both raw and final processed data have been deposited in a public database such as GEO.

☒ Confirm that you have deposited or provided access to graph files (e.g. BED files) for the called peaks.

Data access links *May remain private before publication.*	GEO: GSE109043, PRIDE: PXD013546.
Files in database submission	GSE109043_RAW.tar 353.4 Mb (http)(custom) TAR (of BW)<br>GSE109043_mNSC_SEs_ROSE.table.xlsx 654.0 Kb (ftp)(http) XLSX<br>GSE109043_mNSCs_Carm1_allpeaks.bed.xlsx 241.2 Kb (ftp)(http) XLSX<br>GSE109043_mNSCs_Jmjd1c_allpeaks.bed.xlsx 222.8 Kb (ftp)(http) XLSX<br>GSE109043_mNSCs_Med1_allpeaks.bed.xlsx 595.5 Kb (ftp)(http) XLSX<br><br>1408_MartiQuevedo_01.RAW 134,907 MB =Download<br>1408_MartiQuevedo_02.RAW 99,609 MB =Download<br>1408_MartiQuevedo_02_131114052752.RAW 138,444 MB =Download<br>1408_MartiQuevedo_03.RAW 133,325 MB =Download<br>1408_MartiQuevedo_04.RAW 138,869 MB =Download<br>1408_MartiQuevedo_05.RAW 136,446 MB =Download<br>1408_MartiQuevedo_06.RAW 137,753 MB =Download<br>1408_MartiQuevedo_07.RAW 139,328 MB =Download<br>1408_MartiQuevedo_08.RAW 141,924 MB =Download<br>1408_MartiQuevedo_09.RAW 142,765 MB =Download<br>1408_MartiQuevedo_10.RAW 138,408 MB =Download<br>1408_MartiQuevedo_11.RAW 141,572 MB =Download<br>1408_MartiQuevedo_12.RAW 130,378 MB =Download<br>1408_MartiQuevedo_13.RAW 137,678 MB =Download<br>1408_MartiQuevedo_14.RAW 138,658 MB =Download<br>1408_MartiQuevedo_15.RAW 132,5 MB =Download<br>1408_MartiQuevedo_16.RAW 137,451 MB =Download<br>1408_MartiQuevedo_17.RAW 127,298 MB =Download<br>1408_MartiQuevedo_18.RAW 140,704 MB =Download<br>1408_MartiQuevedo_19.RAW 139,938 MB =Download<br>1408_MartiQuevedo_20.RAW 162,121 MB =Download<br>1408_MartiQuevedo_21.RAW 155,324 MB =Download<br>1408_MartiQuevedo_22.RAW 151,086 MB =Download<br>1529_Marti_Quevedo_01.RAW 76,799 MB =Download<br>1529_Marti_Quevedo_02.RAW 75,688 MB =Download<br>1529_Marti_Quevedo_03.RAW 75,581 MB =Download<br>1529_Marti_Quevedo_04.RAW 73,34 MB =Download<br>1529_Marti_Quevedo_05.RAW 74,096 MB =Download<br>1529_Marti_Quevedo_06.RAW 73,464 MB =Download<br>1529_Marti_Quevedo_07.RAW 72,006 MB =Download<br>1529_Marti_Quevedo_08.RAW 81,895 MB =Download<br>1529_Marti_Quevedo_09.RAW 84,443 MB =Download<br>1604_XL_MartiQuevedo_01.RAW 153,77 MB =Download<br>1604_XL_MartiQuevedo_02.RAW 151,31 MB =Download<br>1604_XL_MartiQuevedo_03.RAW 151,009 MB =Download<br>1604_XL_MartiQuevedo_04.RAW 150,327 MB =Download<br>1604_XL_MartiQuevedo_05.RAW 146,447 MB =Download<br>1604_XL_MartiQuevedo_06.RAW 140,826 MB =Download<br>1604_XL_MartiQuevedo_07.RAW 145,172 MB =Download<br>1604_XL_MartiQuevedo_08.RAW 149,885 MB =Download<br>1604_XL_MartiQuevedo_09.RAW 157,981 MB =Download<br>1604_XL_MartiQuevedo_10.RAW 182,027 MB =Download<br>1604_XL_MartiQuevedo_11.RAW 200,515 MB =Download<br>1604_XL_MartiQuevedo_12.RAW 139,405 MB =Download<br>1604_XL_MartiQuevedo_13.RAW 139,649 MB =Download<br>1604_XL_MartiQuevedo_14.RAW 134,037 MB =Download<br>1604_XL_MartiQuevedo_15.RAW 136,012 MB =Download<br>1604_XL_MartiQuevedo_16.RAW 130,555 MB =Download<br>1604_XL_MartiQuevedo_17.RAW 127,833 MB =Download<br>1604_XL_MartiQuevedo_18.RAW 137,692 MB =Download<br>1604_XL_MartiQuevedo_19.RAW 139,473 MB =Download<br>1604_XL_MartiQuevedo_20.RAW 146,855 MB =Download

1604_XL_MartiQuevedo_21.RAW 172,334 MB =Download
1604_XL_MartiQuevedo_22.RAW 181,858 MB =Download
1604_XL_MartiQuevedo_23.RAW 145,989 MB =Download
1604_XL_MartiQuevedo_24.RAW 145,37 MB =Download
1604_XL_MartiQuevedo_25.RAW 143,108 MB =Download
1604_XL_MartiQuevedo_26.RAW 139,554 MB =Download
1604_XL_MartiQuevedo_27.RAW 140,375 MB =Download
1604_XL_MartiQuevedo_28.RAW 136,282 MB =Download
1604_XL_MartiQuevedo_29.RAW 140,335 MB =Download
1604_XL_MartiQuevedo_30.RAW 149,367 MB =Download
1604_XL_MartiQuevedo_31.RAW 159,556 MB =Download
1604_XL_MartiQuevedo_32.RAW 186,393 MB =Download
1604_XL_MartiQuevedo_33.RAW 192,98 MB =Download
1966_F_MikeDekker_rerun_01.raw 317,308 MB =Download
1966_F_MikeDekker_rerun_02.raw 294,439 MB =Download
1966_F_MikeDekker_rerun_03.raw 445,672 MB =Download
1966_F_MikeDekker_rerun_04.raw 461,449 MB =Download
1966_F_MikeDekker_rerun_05.raw 493,945 MB =Download
1966_F_MikeDekker_rerun_06.raw 410,544 MB =Download
1966_F_MikeDekker_rerun_07.raw 428,781 MB =Download
1966_F_MikeDekker_rerun_08.raw 477,13 MB =Download
1966_F_MikeDekker_rerun_09.raw 446,402 MB =Download
1966_F_MikeDekker_rerun_10.raw 385,947 MB =Download
1966_F_MikeDekker_rerun_11.raw 462,645 MB =Download
1966_F_MikeDekker_rerun_12.raw 526,898 MB =Download
1966_F_MikeDekker_rerun_13.raw 480,582 MB =Download
1966_F_MikeDekker_rerun_14.raw 408,162 MB =Download
1966_F_MikeDekker_rerun_15.raw 407,323 MB =Download
1966_F_MikeDekker_rerun_16.raw 461,737 MB =Download
1966_F_MikeDekker_rerun_17.raw 471,621 MB =Download
1966_F_MikeDekker_rerun_18.raw 394,379 MB =Download
1966_F_MikeDekker_rerun_19.raw 444,811 MB =Download
1966_F_MikeDekker_rerun_20.raw 499,004 MB =Download
1966_F_MikeDekker_rerun_21.raw 403,431 MB =Download
1966_F_MikeDekker_rerun_22.raw 411,258 MB =Download
1966_F_MikeDekker_rerun_23.raw 448,572 MB =Download
1966_F_MikeDekker_rerun_24.raw 476,595 MB =Download
1966_F_MikeDekker_rerun_25.raw 510,072 MB =Download
1966_F_MikeDekker_rerun_26.raw 414,813 MB =Download
1966_F_MikeDekker_rerun_27.raw 505,108 MB =Download
1966_F_MikeDekker_rerun_28.raw

Genome browser session (e.g. UCSC)

No longer applicable

Methodology

Replicates

F-Med15 IP in biological duplicate, Med12 IP is single experiment (in two conditions), ChIP-seq exps are single. Knock-down ChIPs are done in biological duplicate. NSCs stainings are done in technical triplicates.

Sequencing depth

Med1 ChIP-seq: 21122393 reads, 19953973 mapped reads, Carm1 ChIP-seq: 11514203 reads, 11514203 mapped reads, Jmjd1c ChIP-seq; 15573667 reads, 11523003 mapped reads. Control IgG ChIP-seq: 22027163 reads, 20408615 mapped reads. 50 bp single-end reads for all ChIP-seqs.

Antibodies

Jmjd1c antibody (Merck Millipore #17-10262), Carm1 antibody (Cell Signaling Technology #12495), Med1 (Bethyl Labs #A300-793A), IgG (Normal Rabbit IgG: Santa Cruz #sc-2027).

Peak calling parameters

MACS46 v1.4.2 was used for peak calling using default settings, using IgG ChIP-seq as background control

Data quality

Med1 ChIP-seq highlights motifs for NFI, SOX and E-box transcription factors that indeed interact with mediator and colocalise with Mediator on the genome. Med1 ChIP-seq yielded 19956 peaks, Carm1 ChIP-seq yielded 8759 peaks, Jmjd1c ChIP-seq yielded 8099 peaks.

Software

All ChIP-seq data sets were mapped to the mouse mm9 reference genome using Bowtie v0.12.7. MACS46 v1.4.2 was used for peak calling using default settings, using IgG ChIP-seq as background control. Enhancers in mouse NSCs were defined using HOMER, function REGION, and using Bedtools. Super enhancers (SE) were identified using the ROSE algorithm. SE plotting was performed using hockey function in R. Motif analyses were performed using HOMER.
